# Predictive Factors of Visual Outcome in Treatment-Naïve Diabetic Macular Edema: Preliminary Results from the Clinical Study “FOVEA”

**DOI:** 10.3390/jcm12123870

**Published:** 2023-06-06

**Authors:** Rita Serra, Florence Coscas, Jean François Boulet, Diogo Cabral, Thi Ha Chau Tran, Giuliana Solinas, Antonio Pinna, Marco Lupidi, Gabriel Coscas, Fovea Study Group

**Affiliations:** 1 Department of Biomedical Sciences, University of Sassari, 07100 Sassari, Italy; 2 Istituto di Ricerca Genetica e Biomedica (IRGB), CNR, Cittadella Universitaria di Cagliari, 09042 Monserrato, Italy; 3 Centre Ophtalmologique de l’Odéon, 113 bd Saint Germain, 75006 Paris, France; 4 Department of Ophthalmology, Paris VI University, 361 rue Clément Ader, Bâtiment C, 27000 Evreux, France; 5 Instituto de Oftalmologia Dr. Gama Pinto, 1150-255 Lisboa, Portugal; 6 Ophthalmology Department, Lille Catholic Hospitals, Lille Catholic University, INSERM U1172, 59000 Lille, France; 7 Department of Medicine, Surgery, and Pharmacy, Ophthalmology Unit, University of Sassari, 07100 Sassari, Italy; 8 Eye Clinic, Department of Experimental and Clinical Medicine, Polytechnic University of Marche, 60121 Ancona, Italy

**Keywords:** aflibercept, diabetic macular edema, fluorescein angiography, fractal analysis, optical coherence tomography angiography, predictive visual outcome

## Abstract

Diabetic macular edema (DME) is a common cause of vision impairment in diabetic retinopathy. The aim of this study was to analyze the relationship between visual outcome and anatomic changes detected by traditional multimodal retinal imaging and optical coherence tomography angiography (OCTA) in DME eyes under treatment with Aflibercept. Methods: Sixty-six DME eyes of 62 patients under treatment with intravitreal Aflibercept and with one-year follow-up were enrolled. All participants underwent a full ophthalmic evaluation, including best correct visual acuity (BCVA) measurement, spectral-domain optical coherence tomography, fluorescein angiography and OCTA, both at baseline and final examination. Fractal OCTA analysis of the superficial and deep capillary plexus (SCP and DCP) was performed to estimate vascular perfusion density and lacunarity (LAC). Results: At the final examination, there was a significant improvement in terms of BCVA and central macular thickness (CMT). Furthermore, eyes with CMT <373 µm at baseline reached the higher BCVA at the last follow-up. Eyes with CMT ≥373 µm and DCP LAC <0.41 reached a higher final BCVA, if compared with eyes showing the same CMT but higher initial LAC. Conclusion: A 12-month treatment with intravitreal Aflibercept for DME resulted in significant visual and anatomic improvement. Multimodal retinal imaging, together with fractal OCTA analysis, may provide useful biomarkers, predictive of visual outcome in DME.

## 1. Introduction

Although in the last 40 years the risk of blindness secondary to diabetic retinopathy (DR) has decreased, DR still affects about 30% of the individuals with Type 1 or 2 diabetes [1,2].

One of the main causes of vision impairment in DR is diabetic macular edema (DME), a severe complication affecting 20.1% and 25.4% of individuals with Type 1 and 2 diabetes, respectively, during the first 10 years of disease [3].

Even though the exact physiopathological mechanisms are not fully known, some factors seem to play a critical role in DME onset. Noteworthy, chronic hyperglycemia, free oxygen radicals, and the higher levels of vascular endothelial growth factor (VEGF) may synergistically promote vascular leakage and breakdown of the inner blood–retinal barrier, thus leading to DME development with macular thickening and sub- and intra-retinal fluid accumulation [4].

Traditionally, focal laser photocoagulation was the gold standard for DME therapy, as shown by the Early Treatment Diabetic Retinopathy Study (ETDRS) [5]. However, severe complications, including choroidal neovascularization, macular hemorrhage, visual field defects, and contrast sensitivity reduction, have been reported in patients with DME who received focal laser photocoagulation [6].

In 2006, the ophthalmological use of Ranibizumab, an anti-VEGF drug, was approved by FDA, at first for neovascular age-related macular degeneration (AMD) treatment, and later also for DME [4,7].

More recently, Aflibercept, an anti-VEGF agent, has been introduced in the clinical practice as an effective and safe drug for DME management. Aflibercept, a recombination fusion protein, shows a longer half-life and about 100-fold stronger binding affinity to VEGF-A than Ranibizumab. Although both Aflibercept and Bevacizumab are successful in DME treatment, Aflibercept seems to be more effective in terms of visual outcome after one and two years of follow-up, especially in eyes with worse vision at baseline. Furthermore, after the loading phase (five monthly injections), the prescribing information recommends an Aflibercept injection every two months, resulting in a reduced number of injections during the first year of follow-up [8,9,10]. Therefore, Aflibercept is currently considered one of the first line therapeutic approaches in DME [7,11,12].

Although in most DME patients intravitreal anti-VEGF drugs are well-tolerated, safe, and effective, refractory cases have been described [13].

In the last decade, multimodal imaging evaluation and, more recently, optical coherence tomography angiography (OCTA) have allowed the identification of biomarkers predictive of DME development and long-term DR evolution [14,15,16]. Furthermore, fractal OCTA analysis of the superficial and deep capillary plexus (SCP and DCP) has been shown to provide quantitative markers strongly related to DR progression [16].

To our knowledge, we are unaware of any previously published report investigating the application of fractal OCTA analysis to eyes with DME receiving Aflibercept. The purpose of this study was to evaluate the correlation between long-term visual acuity and anatomic changes detected by traditional multimodal retinal imaging and optical coherence tomography angiography (OCTA) in DME eyes under treatment with Aflibercept.

## 2. Materials and Methods

In this prospective multicenter investigation, diabetic patients with treatment-naïve DME were consecutively enrolled from seven high-volume referral centers, members of the FOVEA study group, between February 2017 and March 2018. All the subjects underwent a 12-month follow-up.

The inclusion criteria were: age 18 years and over, diagnosis of diabetes mellitus Type 1 or 2 under treatment with insulin or oral anti-hyperglycemic agents, and presence of treatment-naïve DME.

Exclusion criteria were: history of focal laser treatment, pan-retinal photocoagulation, and cataract or other intraocular surgery within 6 months before enrollment. Furthermore, eyes with ophthalmological diseases other than DR (e.g., AMD, uveitis, retinal vascular occlusions, glaucoma), at baseline or during the follow-up period, were also excluded. Similarly, eyes with low-quality images on traditional retinal imaging or OCTA were excluded.

The current investigation was performed in compliance with the tenets of the Declaration of Helsinki for research involving human subjects and approved by the Paris Review Committee (Protocol no. 2017-A00383-50). Written informed consent to participate was signed by each participant.

### 2.1. Ophthalmologic Examination

All participants underwent extensive ophthalmic evaluation and retinal imaging. BCVA measurement with ETDRS charts, slit-lamp biomicroscopy with dilated indirect fundoscopy, intraocular pressure measurement with Goldmann applanation tonometry, structural OCT, fluorescein angiography (FA), and OCTA were performed.

The presence and entity of DME were assessed by a macular volume scan (49 B-scans within a 30° × 20° area) centered on the fovea using Spectral Domain-OCT (SD-OCT; Spectralis; Heidelberg Engineering, Heidelberg, Germany).

The retinal map analysis protocol of SD-OCT was used to assess retinal thickness in the nine ETDRS sectors. The inner and outer rings were segmented into four quadrants, with radii of 1.5 and 3 mm, respectively, whereas the foveal thickness was defined as the average thickness in the central 1000 μm diameter of the ETDRS grid. Central macular thickness (CMT) was defined as the mean thickness at the point of intersection of six radial scans [17]. Furthermore, the presence of hard exudates, alterations of ellipsoid zone, and distribution of CME (intraretinal vs. sub-retinal fluid) was carefully evaluated on SD-OCT.

The presence of vitreomacular adhesion (VMA) was detected by SD-OCT. Specifically, VMA was defined as the simultaneous presence of perifoveal detachment of the vitreous cortex from the retinal surface together with its attachment at the level of the center of the fovea without alterations of the foveal profile and underlying retinal layers [18].

On the same day, FA (Heidelberg Retina Angiograph II; Heidelberg Engineering, Heidelberg, Germany) using a 55° lens was performed to detect DR features in the eight peripheral fields during early, middle, and late phases.

Spectralis HRA + OCT (Heidelberg Engineering, Heidelberg, Germany) was used to obtain OCTA images. En-face OCT-angiograms were obtained by segmenting a 10° × 10° (3 × 3 mm approximately) volume scan at the level of the ganglion cell layer for SCP and at the inner nuclear layer for DCP. The in-built software (SP-X1701 Update 3, based on Heyex Software Version 1.9.215.0H, Heidelberg Engineering, Heidelberg, Germany) has a projection-artifact removal tool, which automatically removes any shadow-graphic artifact from the selected C-scan. The segmentation strategies have been reported elsewhere [19].

Then, automatic segmentations of both SCP and DCP, automatically generated by the OCTA software (Triton SS-OCT, Topcon, Tokyo, Japan) were carefully checked by an expert retinal specialist (F.C.) and manually adjusted to remove artifacts potentially confounding image interpretation, if needed. Thus, only high-quality images without motion and shadowing artifacts were considered for further analysis.

In case of large cysts involving multi-retinal layers, the offset values of the inner and outer DCP borders were adjusted manually, so that the segmentation lines covered the entire thickness of the inner nuclear and outer plexiform layers. If this manual extension of the DCP range did not resolve segmentation errors with irregularly wavy borders, the flatten band tools were used to move the outer border into the outer nuclear layer, to obtain an accurate measure of the DCP [20].

Then, SCP and DCP OCT-angiograms were exported into a formerly validated custom graphical user interface built in MATLAB (v.r2018a) coding language. The Otsu method was used to binarize the images and the vascular perfusion density (VPD) was calculated. Thereafter, the box-counting method at multiple origins was applied to SCP and DCP images of the binary skeleton to calculate LAC, a quantitative parameter of structural non-uniformity [21,22].

### 2.2. DME Treatment

All DME eyes were treated with five loading doses of intravitreal injections of Aflibercept (2 mg/0.05 mL, Eylea^®^; Bayer Hispania, S.L., Barcelona, Spain) every 4 weeks, as recommended by the prescribing information for Aflibercept [9]. Thereafter, every 8 weeks, extensive ophthalmic evaluation and retinal imaging were performed. Specifically, BCVA measurement with ETDRS charts, slit-lamp biomicroscopy with dilated indirect fundoscopy, intraocular pressure measurement with Goldmann applanation tonometry, and SD-OCT were performed, and then, a new Aflibercept injection was given, if there was a visual impairment secondary to the persistence of fluid or presence of new fluid on OCT evaluation.

Intravitreal Aflibercept was injected after topical anesthesia with lidocaine eye-drops and disinfection of the ocular surface with 5% povidone-iodine. A 30-gauge needle was used to inject Aflibercept 4 mm posterior to the limbus. After injection, slit-lamp biomicroscopy with dilated indirect fundoscopy was performed to exclude the occurrence of any intravitreal-related complication.

### 2.3. Statistical Analysis

Descriptive analysis results are reported as numbers and percentages for categorical variables and as means ± standard deviation (SD) for quantitative variables. After testing the data distribution for normality, *t*-test and Pearson’s test were used, as appropriate. A *p* value < 0.05 was considered statistically significant.

Regression analysis was performed using all parameters evaluated in the present study, in order to identify predictive biomarkers of visual improvement at the last follow-up. The regression tree best fitting to the model was chosen on the basis of the determination coefficient (r^2^ value).

The study data were processed with the Statistical Package for Social Sciences version 20.0 for Mac (IBM, Chicago, IL, USA).

## 3. Results

Sixty-six DME eyes of 62 Caucasian participants (36 men, 26 women; mean age 67 ± 11 years), fully meeting inclusion criteria, were enrolled in this prospective multicenter study. Of them, 27 (15 men, 12 women; mean age 65 ± 13 years) had Type 1 diabetes, whereas the remaining 35 (21 men, 14 women; mean age 68 ± 8 years) had Type 2 diabetes (Table 1).

Mean HbA1c was 7.94 ± 1.22% in the group with the Type 1 diabetes and 7.55 ± 1.01% in the other one (*p* = 0.124).

Mean BCVA at baseline was 64.77 ± 12.73 ETDRS letters, whereas mean CMT was 420.9 ± 103.7 µm. At the last follow-up examination, after 6.07 ± 2.4 intravitreal injections of Aflibercept, there was a significant increase in mean BCVA, which reached 73.01 ± 10.72 ETDRS letters (*p* = 0.004). Overall, after one year of follow-up, mean visual gain was 8.39 ± 12.65 ETDRS letters. Baseline BCVA values were not significantly related to the final visual outcome (r = 0.12, *p* = 0.4).

On baseline FA, 48/66 (72.7%) eyes showed microaneurysms, 49/66 (74.2%) intraretinal microvascular abnormalities (IRMAs), and 22/66 (33.3%) retinal neovascular lesions.

No adverse effects secondary to intravitreal injections (i.e., retinal detachment, central retinal artery occlusion, endophthalmitis) were noted.

At the last follow-up examination, SD-OCT analysis revealed that the number of eyes with macular cysts was halved (31 vs. 63 at baseline; *p* < 0.0001). Furthermore, there was a significant decrease in the number of eyes with subretinal fluid (4 vs. 16 at baseline; *p* = 0.04) and in mean CMT (348.8 ± 104.5 µm vs. 420.9 ± 103.7 µm at baseline; *p* = 0.0005). There was also a significant reduction in the number of eyes with hard exudates (from 22 to 9, respectively; *p* < 0.001) and with ellipsoid zone alterations (from 45 to 29, respectively; *p* < 0.001).

Conversely, no significant differences were observed in terms of OCTA parameters, such as VPD and LAC, between baseline and last follow-up examinations. All quantitative SD-OCT and OCTA data are reported in Table 2.

**Figure 1:**
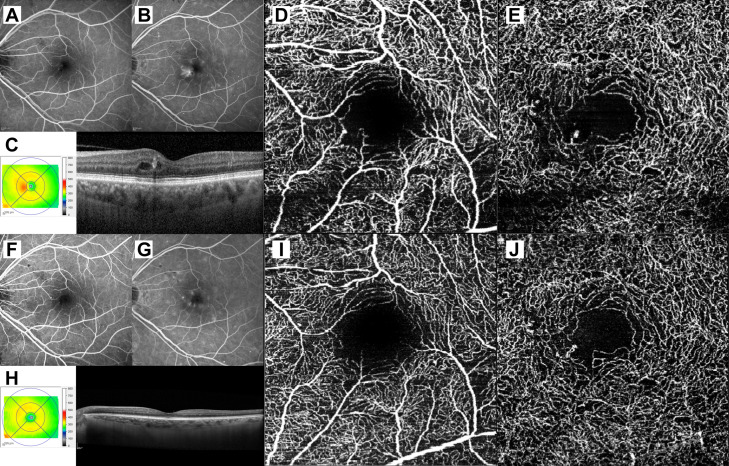
Multimodal imaging and optical coherence tomography angiography of treatment-naïve diabetic macular edema at baseline and 12 months after Aflibercept treatment. (**A**) Early phase of fluorescein angiography (FA) showing hypo- and hyperfluorescent dots corresponding to macular hemorrhages and microaneurysms, respectively. (**B**) Late FA frame discloses a brighter area of hyperfluorescence in the central macula, suggestive of breakdown of the inner blood–retinal barrier and diabetic macula edema, appearing as thickening of the foveal region with intra-retinal cystoid spaces, on spectral domain optical coherence tomography (SD-OCT), at baseline (**C**). (**D**) Optical coherence tomography angiography of DME on the superficial capillary plexus (SCP) and deep capillary plexus (DCP), at baseline (**E**). (**F**,**G**) Early and late FA frames show a complete DME resolution, as confirmed by the SD-OCT scan (**H**) and SCP (**I**) and DCP (**J**) OCTA slabs, 12 months after Aflibercept treatment.

Representative multimodal imaging and OCTA treatment-naïve DME at baseline and 12 months after Aflibercept treatment are shown in Figure 1.

Multiple regression tree models were automatically generated. The regression tree presenting node points VMA, CMT (≥373 μm) and DCP LAC (≥0.41) was chosen, because this model showed the best ability to predict visual improvement at the last follow-up (r^2^ = 0.81).

**Figure 2:**
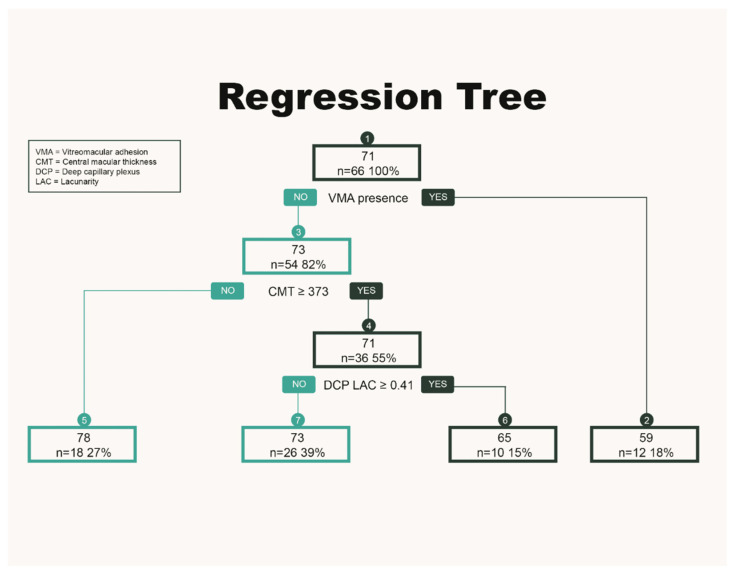
Regression tree predicting final visual outcome in treatment-naïve diabetic macular edema eyes, receiving intravitreal Aflibercept therapy for 12 months.

Specifically, DME eyes with the same initial CMT but different LAC values showed different BCVA at 12-month follow-up. In fact, 36/66 (55%) eyes with CMT ≥ 373 µm presented a final BCVA of 71 ETDRS letters. Of them, 10/36 (27.7%) with DCP LAC ≥ 0.41 had a final BCVA of 65 ETDRS, whereas those with DCP LAC < 0.41 showed a final BCVA of 73 ETDRS letters in 26/36 (72.3%) cases (Figure 2).

## 4. Discussion

In this prospective study including 66 treatment-naïve DME eyes, we investigated the long-term retinal changes after one-year of therapy with Aflibercept. The relationship between BCVA and features on traditional multimodal imaging and OCTA at baseline and after 12 months of follow-up was also assessed.

Overall, our study revealed that mean BCVA significantly improved, reaching 73.01 ± 10.72 ETDRS letters, with a mean visual gain of 8.39 ± 12.65 ETDRS letters (*p* = 0.004). Furthermore, we found that functional improvement was associated with significant reduction of the number of eyes with macular cystoid spaces (from 63 to 31; *p* = 0.0001) and sub-retinal fluid (from 16 to 4; *p* = 0.04), as well as decrease in mean CMT (from 420.9 ± 103.7 to 348.8 ± 104.5 µm; *p* = 0.0005).

Our data are consistent with the results reported by Phase III VISTA-DME and VIVID-DME trials, which assessed cohorts of diabetics with different ethnicities (Caucasian, African American, Asian, etc.), treated with anti-VEGF agents in 42.9% and 8.9% of cases, respectively. Both trials revealed that Aflibercept was well-tolerated and beneficial in DME treatment, simultaneously showing a two-fold or higher improvement of DR severity score [23,24]. These results have been confirmed by the CLARITY study, which also demonstrated the regression of neovascularization in >80% of the eyes under Aflibercept therapy [25].

Likewise, in a real-life study including 29 treatment-naïve DME eyes, Campos Polo et al. [7] found a significant anatomical and functional improvement after one year of intravitreal injections of Aflibercept.

The clinical assessment of DME severity relies mainly on SD-OCT evaluation, which rapidly allows quantification of CMT, a major biomarker of retinal damage. Indeed, clinical evidence indicates that CMT reduction is usually associated with BCVA improvement, and baseline CMT values correlate with long-term visual outcome [26,27].

On the other hand, Bressler et al., in a study including 652 DME eyes treated with intravitreal anti-VEGF drugs, have demonstrated that CMT changes account for only a small part of BCVA changes [28]. Therefore, although SD-OCT provides insights into DME severity, CMT alone cannot be considered as a reliable surrogate for BCVA and a predictive biomarker of visual outcome in DME patients receiving anti-VEGF agents.

Identification of prognostic biomarkers is crucial to improve DME management and patient counseling. There is growing evidence showing that fractal analysis may provide insights into the geometric complexity of branching biological structures, including coronary arterioles, pulmonary bronchi, neoplastic vascular lesions, and retinal vascularity [16,29,30].

Recently, Serra et al. have reported that quantitative fractal OCTA parameters are useful biomarkers to distinguish AMD neovascular lesions characterized by different natural history and prognosis. Moreover, fractal analysis revealed that polypoid CNVs with a different OCTA appearance have similar neovascular architecture and branching complexity [21].

Similarly, Coscas et al. have proposed a predictive model based on fractal OCTA analysis for treatment decision in AMD-related CNV [31].

In our study, fractal OCTA analysis of treatment-naïve DME eyes revealed that mean VPD was 0.36 ± 0.07% both in SCP and DCP. Our VPD data in DME patients are lower than those previously reported by Coscas et al. in healthy controls [32].

DR is a microvasculature disease characterized by capillary changes, such as microaneurysms, capillary closure, and vessel wall alterations, with consequent leakage and DME. Thus, it is not surprising that DME eyes have lower VPD values than healthy controls. Animal studies have demonstrated that in DR eyes, there is an early disappearance of pericytes and endothelial cells, with consequent development of acellular capillaries resulting in vaso-regression and VPD decrease [33]. In this scenario, it has been postulated that glial cell activation promotes the local release of VEGF, which provokes blood–retinal barrier breakdown, capillary leakage, and, finally, DME, appearing on OCTA as hypo-reflective cystoid spaces contributing to VPD reduction [34].

Some authors have suggested that the VPD reduction in DME might reflect a segmentation artifact due to vessel displacement caused by edema pockets [35]. However, this theory is not supported by our results, which show no significant VPD improvement after DME resolution. Presumably, anti-VEGF agents promote anatomical DME improvement with consequent visual gain, but they cannot restore the microvascular DR-induced changes, responsible for DME recurrence when the drug action vanishes. It is likely that the vascular alterations detected in SCP and DCP and quantified by the estimation of fractal parameters may reflect the severity of microvascular DR alterations.

Similarly, in a retrospective observational survey including 84 DR eyes and 14 healthy controls, Kim et al. [15] found a negative correlation between DR severity and VPD. These authors postulated that decreasing capillary density may be associated with worsening DR. Other surveys comparing eyes with and without DME have shown that fractal parameters are correlated with DME. In a prospective study assessing 205 eyes of 129 individuals with a two-year follow-up, SCP VPD was found to be a predictive biomarker of DME occurrence [14].

We are unaware of former investigations exploring the role of LAC, a fractal parameter of structural heterogeneity, in treatment-naïve DME eyes under treatment with intravitreal Aflibercept for 12 months. Our study disclosed that the initial values of CMT, DCP LAC, and the presence/absence of VMA may have a role in predicting the long-term functional outcome in treatment-naïve DME eyes.

The regression tree presented in Figure 2 clearly shows that eyes with VMA present a worse final visual outcome than those without VMA. Although the current literature on this topic is limited, some authors have highlighted that VMA may affect functional and anatomical results of anti VEGF therapy. In fact, VMA may promote a persistent low-grade inflammation associated with the release of inflammatory cytokines, thereby reducing the efficacy of anti-VEGF drugs and, then, the functional results [36,37].

Interestingly, our study disclosed that eyes with the same initial CMT, but different DCP LAC values, have a different visual outcome. Particularly, 10/66 (15%) eyes with an initial CMT ≥ 373 µm and DCP LAC ≥ 0.41 had a final BCVA of 65 ETDRS, whereas 26/66 (39%) of eyes with DCP LAC < 0.41 showed a final BCVA of 73 ETDRS letters.

LAC is a global index of structural non-uniformity. Higher LAC values translate into a greater size distribution of the lacunae and, consequently, a higher degree of “gappiness”. Theoretically, higher LAC values correspond to a more severe form of DME, due to DR-related vascular damage, including increased leakage at level of the DCP, breakdown of the inner blood–retinal barrier and presumably permanent reduction of the capillary network.

Costanzo et al. investigated the predictive role of baseline OCT and OCTA parameters in DME eyes treated with a dexamethasone implant (DEXi). These authors found that statistical models based on the combination of baseline OCT and OCTA parameters may help in the identification of DME eyes responding to a DEXi in the treatment-naïve group [38].

Our study has some intrinsic limitations, including the relatively small sample size and the lack of a control group. Furthermore, we cannot exclude that fractal parameters computed at baseline may be somehow affected by shadowing artifacts mainly related to the presence of large cysts involving multi-retinal layers. On the other hand, to our knowledge, this is the first report on the application of fractal OCTA analysis, with LAC computation, to treatment-naïve DME eyes receiving intravitreal Aflibercept for 12 months.

In conclusion, our study confirms that Aflibercept is a successful first line treatment for DME. Furthermore, our results suggest that fractal OCTA analysis, in conjunction with SD-OCT, provides quantitative parameters, which may be potential predictors of long-term visual outcome in DME under treatment with Aflibercept.

## Figures and Tables

**Table 1:** Demographic characteristics of treatment-naïve patients with diabetic macular edema, at baseline.

Total eyes, *n* (%)	66
Total patients, *n* (%)	62
Sex	
- Male, *n* (%)	36 (58%)
- Female, *n* (%)	26 (42%)
Age, mean ± SD (years)	67 ± 11

Categorical variables are presented as *n* (%). Continuous variable is presented as mean ± standard deviation (SD).

**Table 2:** Comparison of quantitative spectral-domain optical coherence (SD-OCT) and OCT Angiography (OCTA) parameters at baseline and last follow-up, in treatment-naïve diabetic macular edema eyes receiving intravitreal Aflibercept for 12 months.

	Baseline	12-Month Follow-Up	*p* Value
BCVA, mean ± SD (ETDRS letters)	64.77 ± 12.73	73.01± 10.72	0.004
SD-OCT			
- MT (μm)	420.9 ± 103.7	348.8 ± 104.5	0.0005
OCTA SCP			
- VPD mean ± SD (%)	0.36 ± 0.07	0.39 ± 0.06	>0.05
- LAC mean ± SD	0.42 ± 0.22	0.37 ± 0.05	>0.05
OCTA DCP			
- VPD mean ± SD (%)	0.36 ± 0.07	0.38 ± 0.07	>0.05
- LAC mean ± SD	0.40 ± 0.15	0.39 ± 0.16	>0.05

Continuous variables are presented as mean ± standard deviation (SD). BCVA = Best-corrected visual acuity; CMT = Central macular thickness; SCP = Superficial capillary plexus; DCP = Deep capillary plexus; VPD = Vascular Perfusion Density; LAC = Lacunarity.

## Data Availability

Not applicable.

## Appendix A

The FOVEA study group Members.

Participants of the FOVEA Study Group included the following:

Pierre-Loic Cornut (Clinique du Val d’Ouest—Centre Pole Vision 39 chemin de la Vernique, 69130 ECULLY, France), Joel Uzzan (Clinique Mathilde de Rouen, Rouen, France), Flore DeBats (Ophthalmology Department, Hôpital de La Croix Rousse, Lyon, France), Jean-Philippe Theron (Institut ophtalmique de Somain, 28 rue Anatole 59490 Somain, France), Benjamin Wolff (Centre ophtalmologique Maison Rouge. 6, rue de l’église, 67000 Strasbourg, France), Catherine Francais (Centre Ophtalmologique de l’Odéon, 113 bd Saint Germain, Paris, France), Catherine Favard (Centre Ophtalmologique de l’Odéon, 113 bd Saint Germain, Paris, France; Ophthalmology Department, Hôpital Cochin, Paris, France).
